# Treatments for Shoulder Impingement Syndrome: A PRISMA Systematic Review and Network Meta-Analysis

**DOI:** 10.1097/MD.0000000000000510

**Published:** 2015-03-13

**Authors:** Wei Dong, Hans Goost, Xiang-Bo Lin, Christof Burger, Christian Paul, Zeng-Li Wang, Tian-Yi Zhang, Zhi-Chao Jiang, Kristian Welle, Koroush Kabir

**Affiliations:** From the Department of Orthopedic and Trauma Surgery (WD, Z-LW, T-YZ), Central Hospital of PetroChina, Langfang, Hebei, China; Department of Orthopedic and Trauma Surgery (WD, CB, KW, KK), University Hospital Bonn, Bonn; Department of Orthopedic and Trauma Surgery (HG), Hospital Wermelskirchen, Wermelskirchen, Germany; Department of Orthopedic and Trauma Surgery (X-BL), Rizhao People's Hospital, Rizhao, Shandong, China; Department of Orthopedic and Trauma Surgery (CP), Evangelic Wald-Krankenhaus, Bonn, Germany; and Department of Fundamental Science (Z-CJ), North China Institute of Aerospace Engineering, Langfang, Hebei, China.

## Abstract

Many treatments for shoulder impingement syndrome (SIS) are available in clinical practice; some of which have already been compared with other treatments by various investigators. However, a comprehensive treatment comparison is lacking.

Several widely used electronic databases were searched for eligible studies. The outcome measurements were the pain score and the Constant–Murley score (CMS). Direct comparisons were performed using the conventional pair-wise meta-analysis method, while a network meta-analysis based on the Bayesian model was used to calculate the results of all potentially possible comparisons and rank probabilities.

Included in the meta-analysis procedure were 33 randomized controlled trials involving 2300 patients. Good agreement was demonstrated between the results of the pair-wise meta-analyses and the network meta-analyses. Regarding nonoperative treatments, with respect to the pain score, combined treatments composed of exercise and other therapies tended to yield better effects than single-intervention therapies. Localized drug injections that were combined with exercise showed better treatment effects than any other treatments, whereas worse effects were observed when such injections were used alone. Regarding the CMS, most combined treatments based on exercise also demonstrated better effects than exercise alone. Regarding surgical treatments, according to the pain score and the CMS, arthroscopic subacromial decompression (ASD) together with treatments derived from it, such as ASD combined with radiofrequency and arthroscopic bursectomy, showed better effects than open subacromial decompression (OSD) and OSD combined with the injection of platelet-leukocyte gel. Exercise therapy also demonstrated good performance. Results for inconsistency, sensitivity analysis, and meta-regression all supported the robustness and reliability of these network meta-analyses.

Exercise and other exercise-based therapies, such as kinesio taping, specific exercises, and acupuncture, are ideal treatments for patients at an early stage of SIS. However, low-level laser therapy and the localized injection of nonsteroidal anti-inflammatory drugs are not recommended. For patients who have a long-term disease course, operative treatments may be considered, with standard ASD surgery preferred over arthroscopic bursectomy and the open surgical technique for subacromial decompression. Notwithstanding, the choice of surgery should be made cautiously because similar outcomes may also be achieved by the implementation of exercise therapy.

## INTRODUCTION

Shoulder pain is a common presenting complaint from patients of all ages in daily clinical practice, affecting approximately one-third of individuals during their lifetime.^1^ Such pain may lead to the impairment of shoulder joint function and to severe reduction in quality of life. Shoulder impingement syndrome, which is defined as the compression of the rotator cuff and the subacromial bursa, is considered to be one of the most common causes of shoulder pain and may be cited as a contributing factor to shoulder pain in up to 65% of cases.^2^ The typical sign of SIS is pain localized to the anterolateral acromial area, which may also radiate to the lateral midhumerus. Pain at night is another important complaint in these patients. Concurrently, a general loss of muscle strength may be noted.^3^

Neer graded SIS into 3 different stages.^4^ In stage I, the typical characteristics are reversible lesions with edema and hemorrhage; most patients younger than 25 years are in this category. In stage II, chronic inflammation or repeated episodes of impingement lead to histomorphological changes, such as fibrosis and thickening of the supraspinatus, the long biceps tendon, and subacromial bursae. Patients in this stage are usually between 25 and 40 years of age. In stage III, in patients more than 40 years of age, tears of the rotator cuff, rupture of the biceps tendon, and bony changes may be observed, accompanied by significant tendon degeneration following a long history of refractory tendinitis.

The main goals of SIS treatments are to relieve pain and to solve the mechanical problem causing the functional impairment. The SIS treatment strategy varies according to disease stage. At an early stage of SIS, which usually refers to stage I or early stage II, some nonoperative treatments may be effective, such as muscle exercises, for example, the training of the periscapular muscles (pectoralis minor, trapezius, serratus, and rhomboids) and strengthening of the rotator cuff (supraspinatus, infraspinatus, teres minor, and subscapularis), which functions as the stabilizer of the shoulder joint. Some investigators have also reported on many other nonoperative treatment methods, such as pulsed electromagnetic field therapy,^5,6^ manual therapy,^7–10^ kinesio taping therapy,^11,12^ localized drug injection of corticosteroids, hyaluronate, or NSAIDs,^11–18^ diacutaneous fibrolysis therapy,^19^ specific exercise therapy that includes concentric and eccentric exercises for the scapula stabilizers and dynamic humeral centering and scapular stabilization exercises,^20–22^ microwave diathermy therapy,^23^ ultrasound therapy,^24^ low-level laser therapy,^24–28^ radial extracorporeal shockwave therapy,^29^ and acupuncture therapy.^30^ After these treatments have been performed, some patients may be relieved of SIS. However, for other patients, operative treatment should be considered. The most prevalent surgical methods are ASD and OSD.^31–37^ Additionally, some adjustments have been made based on these 2 classic techniques, for example, arthroscopic bursectomy,^36^ ASD combined with radiofrequency therapy, and OSD combined with localized platelet-leukocyte gel injection (PLG).^38,39^

However, the abundance of treatment choices do not necessarily facilitate the physician's decision making but rather indicates that no consensus exists regarding which treatment options are suitable. Many RCTs have been conducted to compare the effectiveness of different treatments, supporting certain conclusions. Some systematic reviews have also been published that concentrated only on the pair-wise comparison of different treatments, but no review including all of the available treatments has been conducted. Due to the limitations of the existing reviews and the fact that many relatively new studies have been published, a prominent need exists to conduct an accurate and comprehensive review of this topic.

Network meta-analysis enables comparisons of the effectiveness of all treatments considered. Furthermore, the statistical method based on Bayesian theory enables calculation of the rank probability for each treatment.^40^ In this type of analysis, investigators may consider all of the possible relevant treatments. Clearly, this approach is in accordance with actual situations in daily clinical practice.^41^

In this review, we have endeavored to provide useful information regarding comparisons among all treatments for SIS. We hope that the results will aid physician decision making.

## MATERIALS AND METHODS

### Eligibility Criteria

This study was based on the Preferred Reporting Items for Systematic Reviews and Meta-Analyses (PRISMA) statement.^42^ Since this study was a review of published studies, ethical approval was not required. Randomized controlled trials that included all of the following criteria were considered eligible: adults older than 18 years; a diagnosis of SIS, not caused by any other systemic disease or acute trauma; the evaluation of at least 2 SIS interventions, including placebo or sham treatment; reported results of pain relief or functional recovery; and reported results after at least 2 weeks of follow-up.

### Search Strategy

Medline, Embase, and the Cochrane Central Register of Controlled Trials (CENTRAL) were searched from the inception of each database to 15 April 2014. The Medline and Embase databases were searched together via www.embase.com (Elsevier, The Netherlands). The search was conducted using the keywords shoulder, subacrom∗, supraspinat∗, rotator cuff, and impingement, and it was limited to RCTs (List 1). Additionally, all of the available reviews related to SIS treatments were manually screened for any additional possibly relevant studies. No language limit was applied.

List 1 Search Strategy used in www.embase.com (step by step):

1. #1 shoulder OR ‘shoulder’/exp
2. #2 ‘rotator cuff’ OR ‘rotator cuff’/exp
3. #3 subacrom∗
4. #4 supraspinat∗
5. #5 #1 OR #2 OR #3 OR #4
6. #6 impingement
7. #7 #5 AND #6
8. #8 ‘shoulder impingement syndrome’/exp
9. #9 #7 OR #8
10. #10 random∗:ab,ti OR factorial∗:ab,ti OR crossover∗:ab,ti OR placebo∗:ab,ti OR control∗:ab,ti OR trial:ab,ti OR group∗:ab,ti OR ‘crossover procedure’/exp OR ‘single blind procedure’/exp OR ‘double blind procedure’/exp OR ‘randomized controlled trial’/exp
11. #11 #9 AND #10.

### Study Selection

Two independent reviewers (WD and X-BL) screened the title and abstract of the retrieved articles, and the full text was reviewed as necessary. The studies that were potentially relevant according to the eligibility criteria were selected. Disagreements regarding study inclusion were resolved by discussion, and in cases of persistent disagreement, a third reviewer (Z-LW) was consulted.

### Data Extraction

Two independent reviewers (WD and X-BL) conducted the data extraction. In cases where the author provided more than 1 follow-up data point, the time point closest to 12 months was adopted. The data were then integrated by WD. Discrepancies between the 2 data extraction results were reviewed by WD and were then resolved by discussion. Similarly, a third reviewer (T-YZ) was consulted if agreement could not be reached between these 2 reviewers.

The evaluation of the primary outcome of pain score was performed based on the visual analog scale (VAS) pain score, the numerical rating scale (NRS) pain score, and the Likert pain score. The original values of these pain scores were then *adjusted to* the range of 0 to 10 (0 for no pain and 10 for the worst imaginable pain).

The secondary outcome of the CMS encompassed subjective (pain and daily activities) and objective (range of motion and strength) assessments (range from 0 to 100, with higher scores being better).

Because patients who underwent surgery usually had a worse condition and a longer disease course than those who were treated nonoperatively, as well as because most of them had already undergone nonoperative treatments at an earlier time, we separated the studies into 2 subgroups according to their focus on nonoperative treatments or operative treatments. These 2 subgroups were then analyzed. In some studies, exercise therapy was compared with surgical treatments; in these cases, the studies were absorbed into the operative treatment subgroup. Interventions employing the same principles but different approaches were assigned the same treatment name. Finally, the interventions were grouped into 20 treatment strategies; some of which represented combinations of 2 treatments. The Cochrane Risk of Bias Tool in RevMan (Review Manager, Version 5.2; Copenhagen: The Nordic Cochrane Centre, The Cochrane Collaboration) was then utilized to determine the quality of the RCTs included.

### Statistical Analysis

First, the pair-wise meta-analysis was conducted using a random-effects model. The results of the studies that compared the same pair of treatments were synthesized. The results are reported as the mean difference (MD) with corresponding 95% confidence interval (CI). All calculations were performed using STATA (Version 12.0, Stata Corporation, College Station, TX).

Second, a random-effects network model was built within the Bayesian framework using the Markov Chain Monte Carlo (MCMC) algorithm in WinBUGS (Bayesian inference Using Gibbs Sampling for Windows, Version 1.4.3; Imperial College and MRC, UK), which is freely available statistical software that is based on the MCMC algorithm.^43^ The changes in the pain score and the CMS in each study were used to compose the networks. Altogether, 4 networks were built as follows: pain score change associated with nonoperative treatments (network 1), CMS change associated with nonoperative treatments (network 2), pain score change associated with operative treatments (network 3), and CMS change associated with operative treatments (network 4). A 95% CI of the MD beyond the null value was considered to indicate statistical significance. Four Markov chains were run for 40,000 iterations simultaneously. A thinning interval of 10 was applied, indicating that 1 sample was collected every 10 iterations. The first 10,000 iterations were considered as burn-in iterations, and no sample was collected during this period because these iterations may have been affected by the arbitrary values assigned at the starting point of each chain. The Brooks–Gelman–Rubin method was used to assess convergence.^44^ By this process, a potential scale reduction factor (PSRF) was calculated by comparing within-chain and between-chain variance. A PSRF very close to 1 was considered to indicate an approximate convergence. The probability of rank for each treatment was also estimated by calculating the MD compared with that of any other treatments. Then, the rank probability data were imported into STATA, which then produced surface plots under the cumulative ranking (SUCRA) curve.^45^

### Inconsistency Analyses

Next, a Z test was performed to examine the inconsistency of the model.^46^ If a loop existed in the network (eg, A-B-C), each comparison in this loop (eg, A vs C) may have conferred an indirect value derived from other comparisons in the loop (eg, A vs B and B vs C), and this indirect value was compared with its direct value. Then, the Z value and its corresponding *P* value were calculated, and if the *P* value was >0.05, no statistically significant difference was noted. The results of the indirect comparisons were analyzed by ITC (Indirect Treatment Comparison, Version: 3.0; Canadian Agency for Drugs and Technologies in Health, Canada).

### Sensitivity Analyses and Meta-Regression

Finally, sensitivity analyses were conducted by excluding the low-quality studies, which contained <3 low risk items in the Cochrane Risk of Bias Tool. The rank probabilities were again calculated. If there was no significant change, the outcome of the meta-analysis was considered to be reliable.

Additionally, a meta-regression was performed to ascertain the relationship between the sample size and the treatment effect using the method recommended by the UK's National Institute for Health and Care Excellence.^47^ A single interaction term was used as the covariate. Moreover, the deviance information criterion (DIC) was used as the measure of model fit. A lower DIC value was preferred because it suggested a more parsimonious model.^48^ If the covariate was associated with the result, there should be a significant reduction of the DIC, and the 95% CI of the regression coefficient for the covariate should not cover the null value.

## RESULTS

### Eligible Studies

**FIGURE 1:**
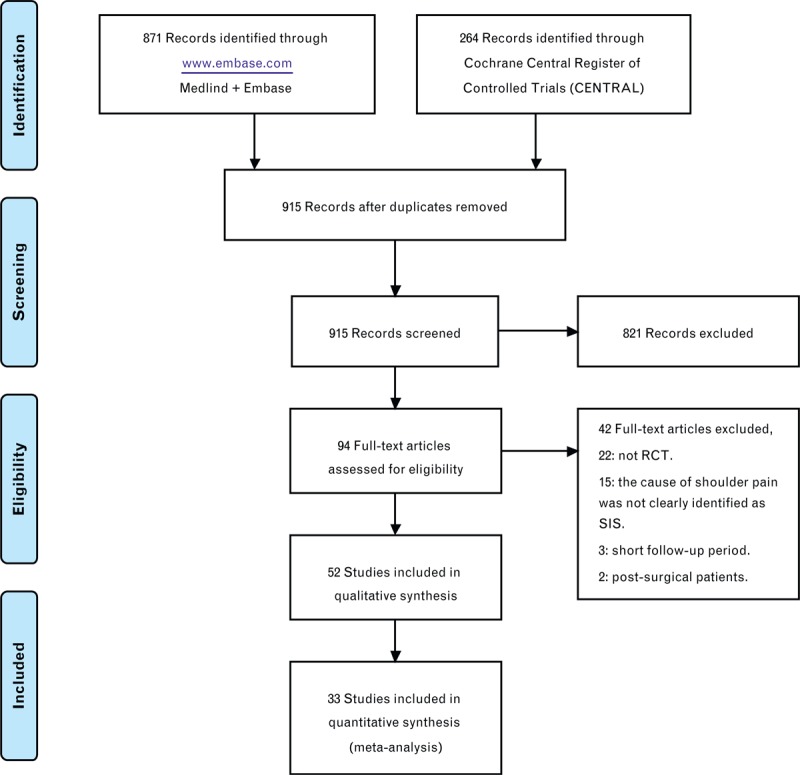
PRISMA 2009 flow diagram.

According to the search strategy, 915 records were identified. After the titles and abstracts were screened, a total of 94 records were screened for eligibility by full-text review. After careful full-text screening, 42 articles were rejected due to the reasons listed in Figure 1, and the remaining 52 articles were entered into the qualitative synthesis procedure. Of these 52 articles, 4 articles^49–52^ were focused on treatments that did not match treatments in other articles, 1 article^35^ was derived from an included study but reported different follow-up results, 6 articles^33,53–57^ used outcome measurements other than the pain score and CMS, and the pain score or CMS results were reported in the other 8 articles,^34,37,58–63^ but the articles were not suitable for statistical analysis. The findings from these articles were also included in the discussion section. Finally, 33 RCTs were included in the quantitative synthesis procedure. The networks of nonoperative treatments included 28 studies (26 reported the pain score and 12 reported the CMS), whereas the networks of operative treatments included 5 studies (5 reported the pain score and 3 reported the CMS). A total of 2300 patients were included in the studies, 2065 of whom received nonoperative treatments and 235 of whom underwent operative treatments. The following standardized headings were extracted: authors, publication years, interventions, number of patients, outcome measures, follow-up time points, and results (Table 1).

**TABLE 1:**
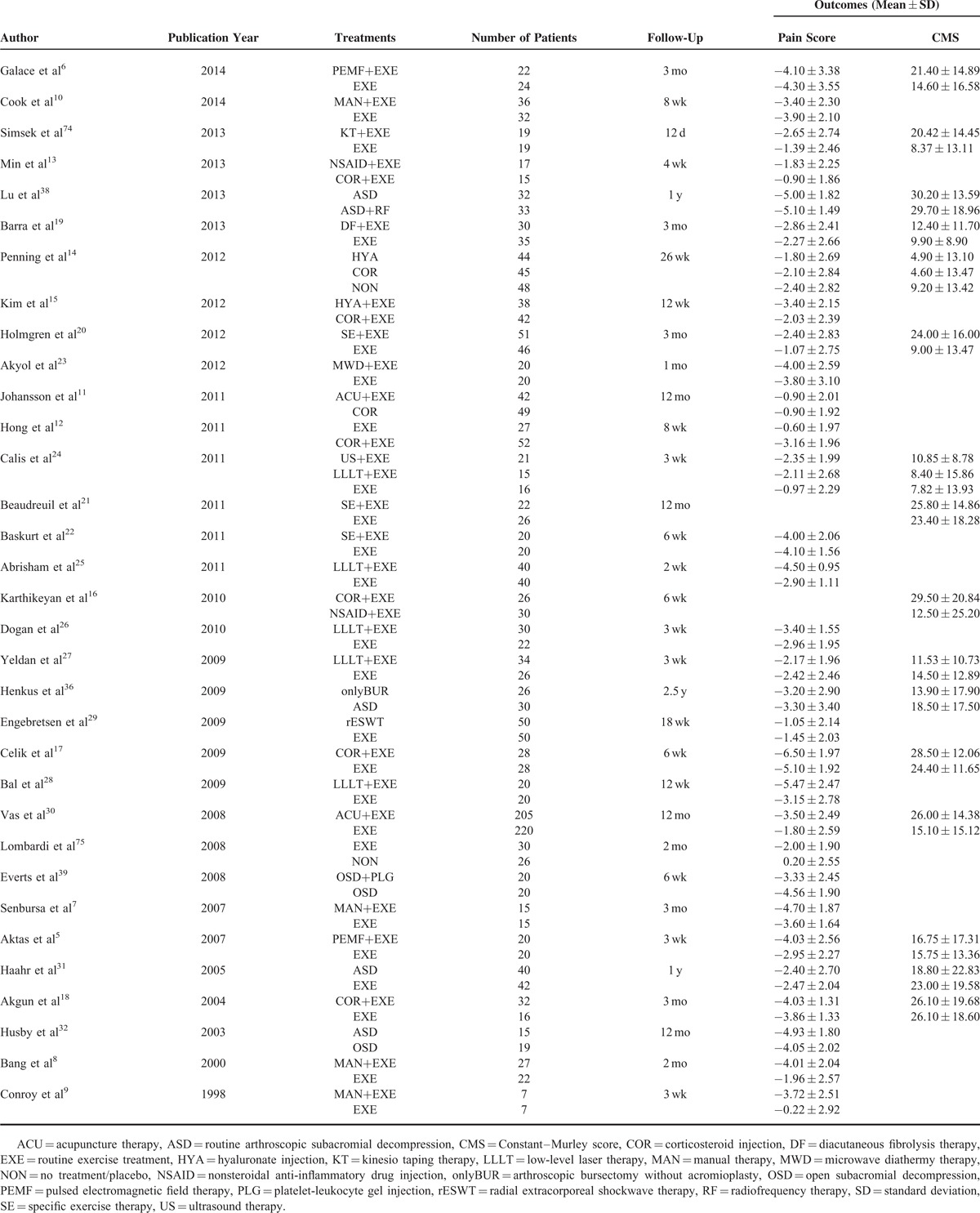
Data of Included Studies

**FIGURE 2:**
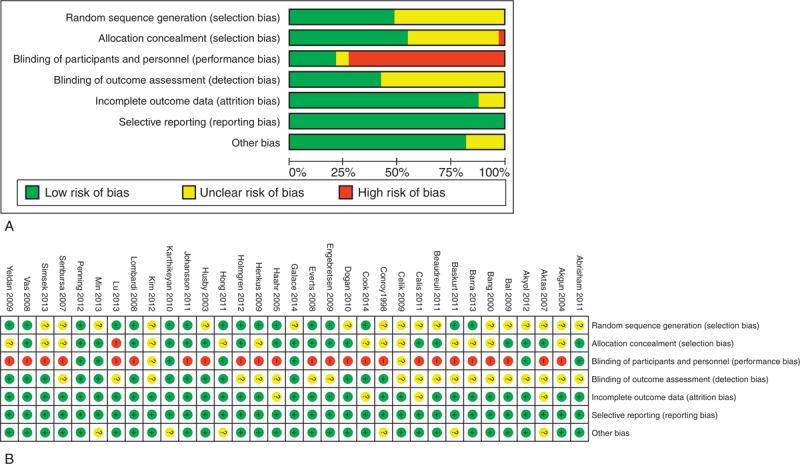
(A) Risk of bias graph. (B) Risk of bias summary.

The results of bias risk for the included RCTs are shown in Figure 2. All the studies were described as “randomized.” However, only 16 of them reported the details of randomization, and allocations were properly concealed in 18 of them. As the clinical involved many different treatments, the blinding of treatment performance appeared difficult. In the performance bias examination, only 7 RCTs were low risk, while 24 RCTs were high risk. Moreover, the blinding of outcome assessment was clearly described in only 14 of the 33 studies.

### Pair-Wise Meta-Analysis

All data, which were suitable for conventional pair-wise meta-analysis, were entered into STATA, and random-effects models were developed. Then, the MDs and 95% CIs were calculated. Regarding nonoperative treatments, 19 pairs of pain score comparisons were performed. Four pairs had 95% CIs beyond the null value, which were considered to represent significant differences, as follows: ACU+EXE versus EXE (MD −1.70, 95% CI −2.18 to −1.22), COR+EXE versus HYA+EXE (MD 1.37, 95% CI 0.38–2.36), EXE versus LLLT+EXE (MD 1.01, 95% CI 0.15–1.87), and EXE versus NON (MD −2.20, 95% CI −3.39 to −1.01). No significant difference was detected in the remaining 15 comparisons. Regarding the CMS, 3 of 10 pairs of comparisons had 95% CIs beyond the null value as follows: ACU+EXE versus EXE (MD 10.90, 95% CI 8.10–13.70), EXE versus KT+EXE (MD −12.05, 95% CI −20.82 to −3.28), and COR+EXE versus NSAID+EXE (MD 17.00, 95% CI 4.94–29.06), with the remaining 7 pairs showing no significant differences. Regarding operative treatments, none of the comparisons (4 pairs for pain score and 2 pairs for CMS) showed significant differences. The results are shown in the upper triangle of Tables 2–5, and the significant differences are shaded.

**TABLE 2:**
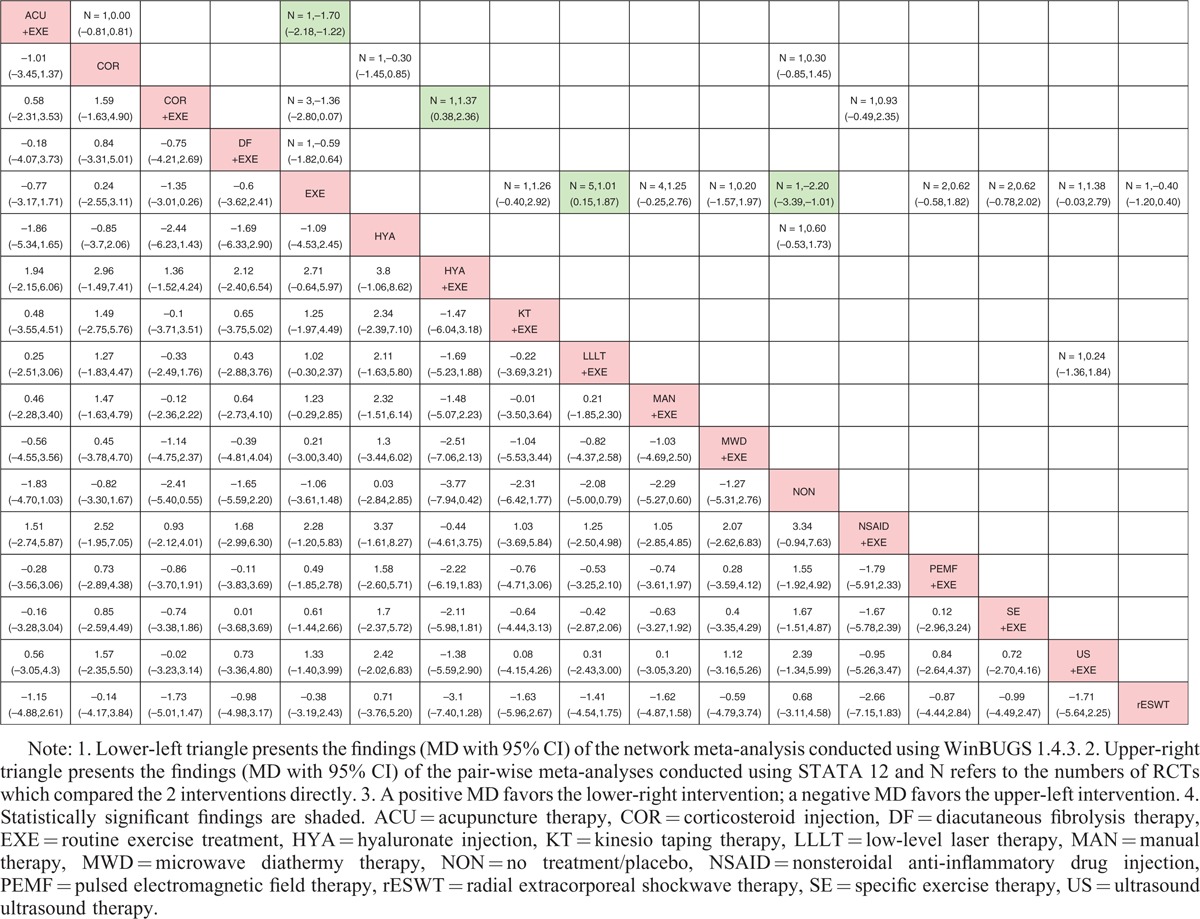
Results of Conservative Treatments (Pain Score)

**TABLE 3:**
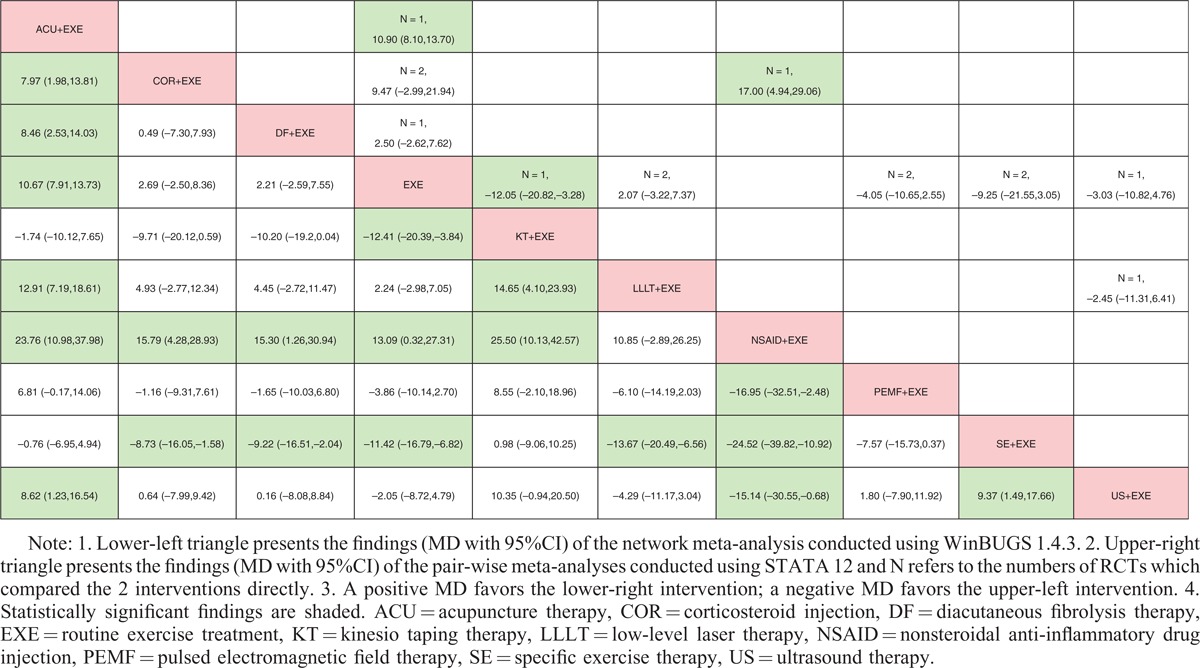
Results of Conservative Treatments (CMS)

**TABLE 4:**
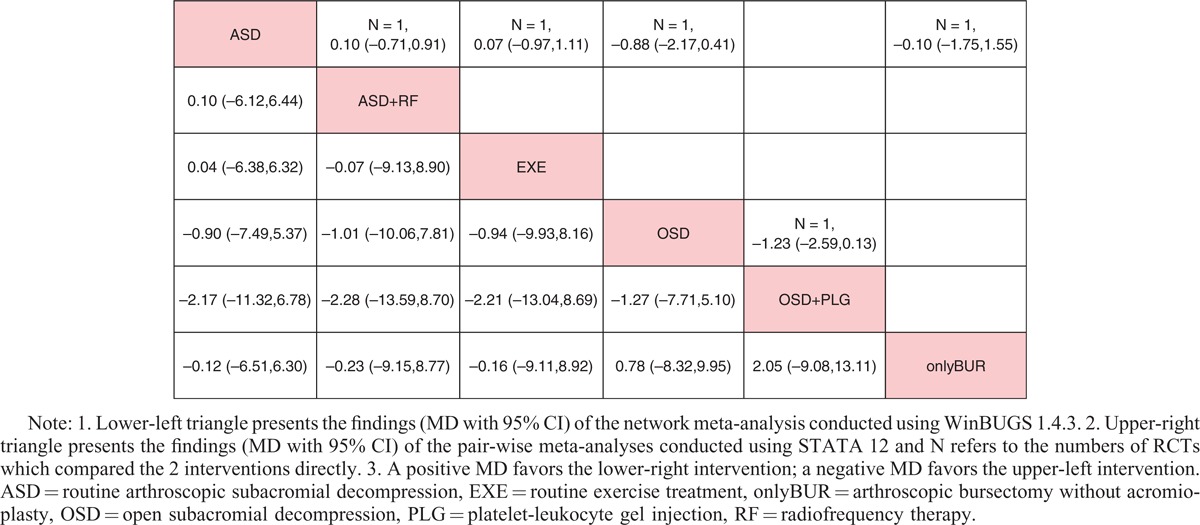
Results of Operative Treatments (Pain Score)

**TABLE 5:**
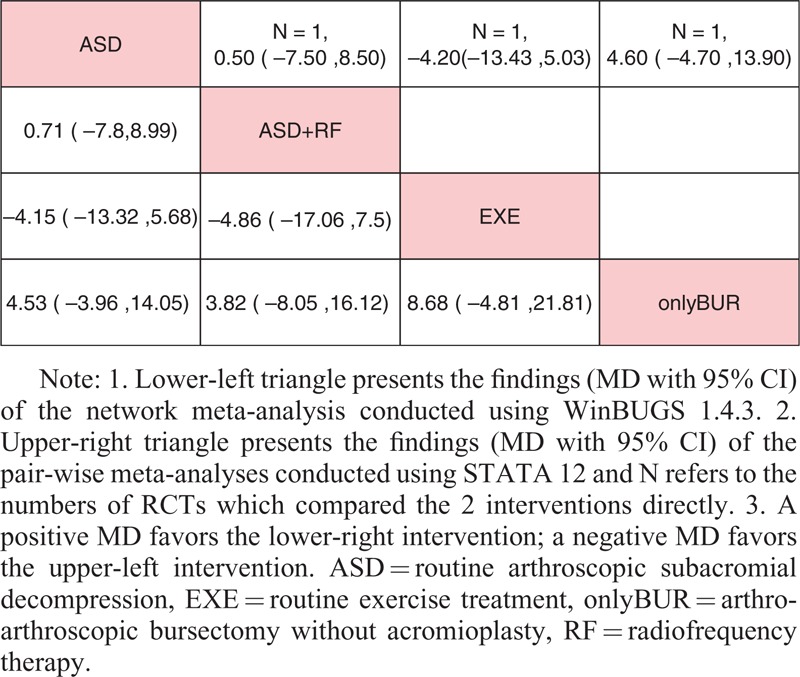
Results of Operative Treatments (CMS)

### Network Meta-Analysis

**FIGURE 3:**
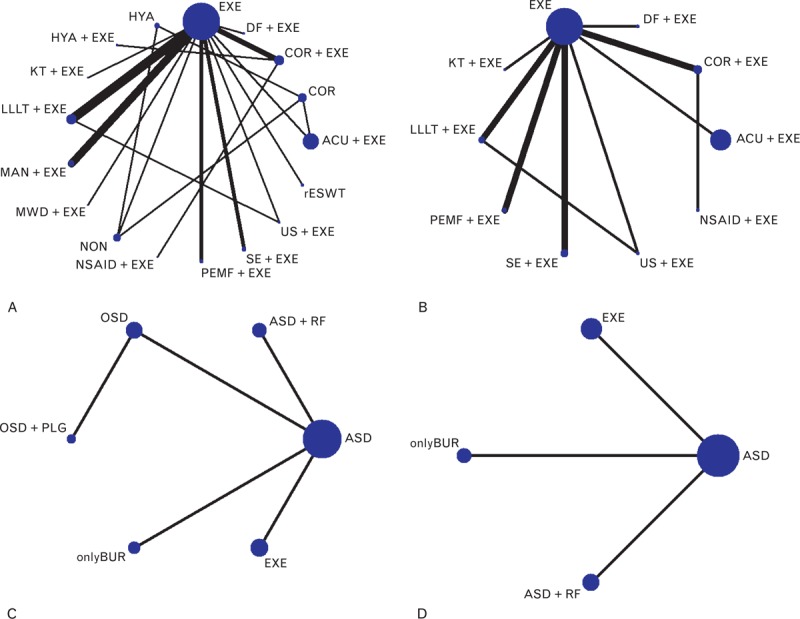
(A) Network 1: Nonoperative treatments (pain score). (B) Network 2: Nonoperative treatments (CMS). (C) Network 3: Operative treatments (pain score). (D) Network 4: Operative treatments (CMS). Note: the size of the circle represents the number of patients; the thickness of the edge represents the number of studies. ACU = acupuncture therapy, ASD = routine arthroscopic subacromial decompression, COR = corticosteroid injection, DF = diacutaneous fibrolysis therapy, EXE = routine exercise treatment, KT = kinesio taping therapy, LLLT = low-level laser therapy, NSAID = nonsteroidal anti-inflammatory drug injection, OSD = open subacromial decompression, PEMF = pulsed electromagnetic field therapy, PLG = platelet-leukocyte gel injection, RF = radiofrequency therapy, SE = specific exercise therapy, US = ultrasound therapy.

These direct comparisons were then combined into 4 comprehensive networks (Figure 3, the size of the circle represents the number of patients, and the thickness of the edge corresponds to the number of studies). All the differences of possible comparisons, including the potential comparisons, were calculated, and the MDs and 95% CIs were obtained. The iterations showed good convergence, as revealed by the strong linearity in the graphical diagnostic plots. Moreover, the PSRFs of parameters were all unlimitedly close to 1 without exception, which also represented good convergence. The results are listed in the lower triangle of Tables 2–5, and significant differences are shaded.

Regarding the nonoperative treatments, no significant difference was found in the outcome of the pain score network analysis, that is, 4 pairs of comparisons (ACU+EXE vs EXE, COR+EXE vs HYA+EXE, EXE vs LLLT+EXE, and EXE vs NON) exhibited different results than those of the pair-wise meta-analysis mentioned above. However, with respect to the CMS, the network meta-analysis showed better concordance with the conventional pair-wise meta-analysis. Most of the results were the same except for those of the comparison of EXE versus SE+EXE, which demonstrated a significant difference in the network comparison (MD −11.42, 95% CI −16.79 to −6.82) but not in the conventional comparison (MD −9.25, 95% CI −21.55 to 3.05).

Regarding operative treatments, the results of both the network comparisons and pair-wise comparisons of the pain score and CMS also showed no significant differences.

**FIGURE 4:**
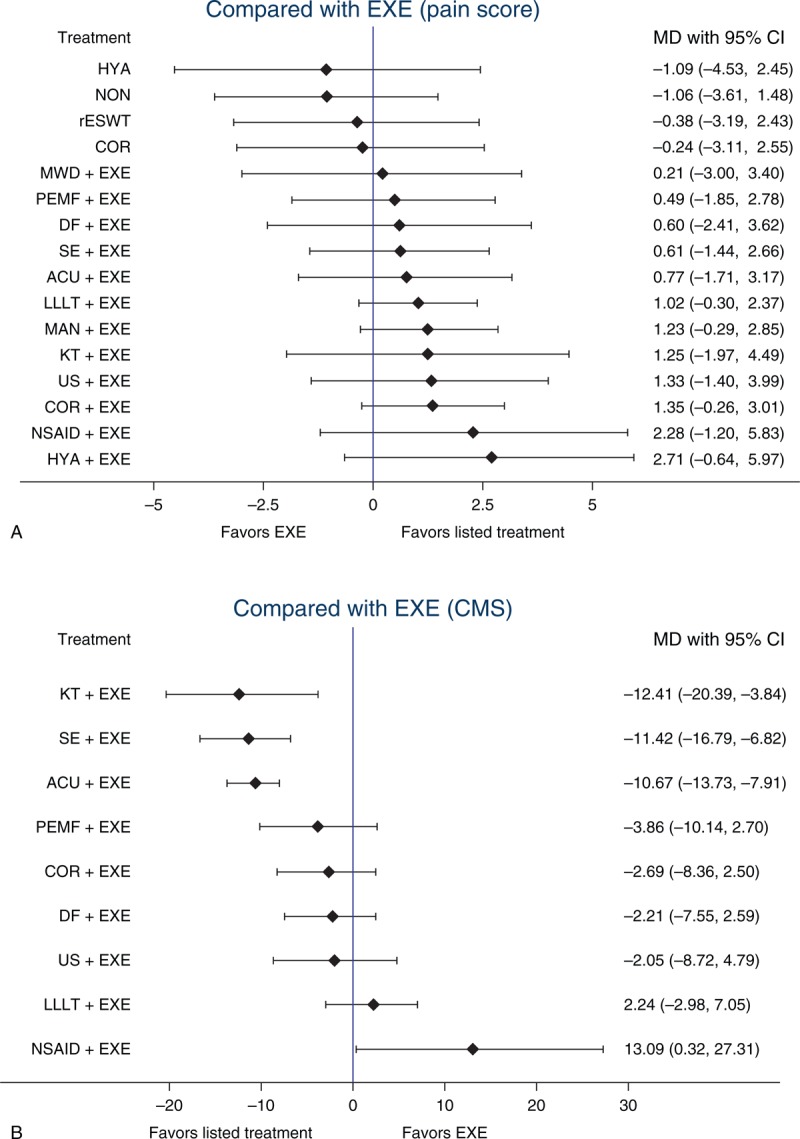
(A) Treatments compared with EXE (pain score). (B) Treatments compared with EXE (CMS). ACU = acupuncture therapy, COR = corticosteroid injection, DF = diacutaneous fibrolysis therapy, EXE = routine exercise treatment, HYA = hyaluronate injection, KT = kinesio taping therapy, LLLT = low-level laser therapy, MAN = manual therapy, MWD = microwave diathermy therapy, NON = no treatment/placebo, NSAID = nonsteroidal anti-inflammatory drug injection, PEMF = pulsed electromagnetic field therapy, rESWT = radial extracorporeal shockwave therapy, SE = specific exercise therapy, US = ultrasound therapy.

Because EXE was the most commonly used treatment in clinical practice, a series of comparisons between other nonoperative treatments and EXE were performed. Regarding pain score, no treatment exhibited a significant difference compared with EXE; however, EXE demonstrated a trend toward better results than the treatments that did not contain EXE, such as COR, HYA, rESWT, and NON. If another therapy were added to EXE, a better effect may be achieved. With regard to CMS, a similar outcome was obtained with most treatments that were composed of EXE and another therapy exhibiting a better treatment effect than EXE alone, except for LLLT+EXE and NSAID+EXE. Among these treatments, KT+EXE, SE+EXE, ACU+EXE, and NSAID+EXE had different effects with significant differences observed. These results are presented in 2 forest plots (Figure 4).

### Rank Probability

**FIGURE 5:**
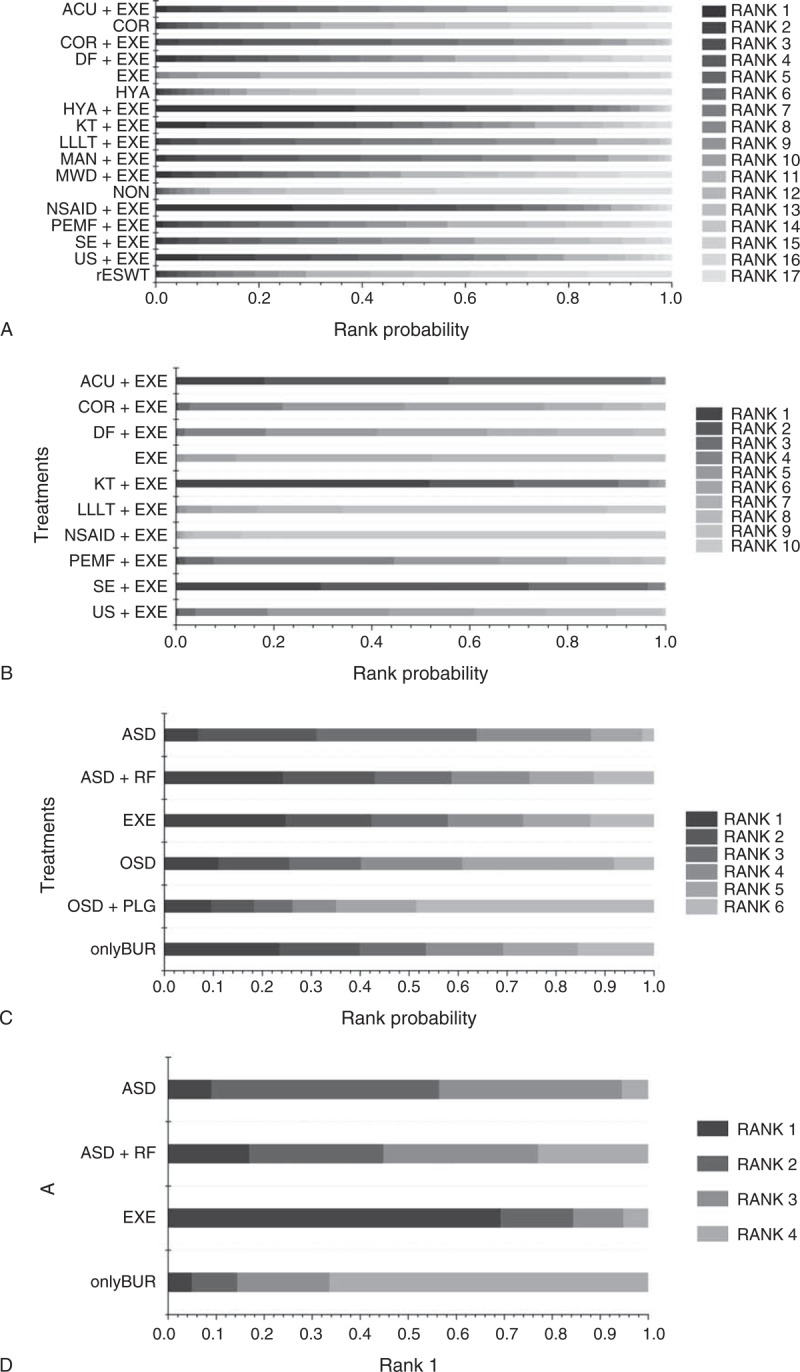
(A) Rank probability of nonoperative treatments (pain score). (B) Rank probability of nonoperative treatments (CMS). (C) Rank probability of operative treatments (pain score). (D) Rank probability of operative treatments (CMS). Note: 1. Different gray scales represent different ranks, with rank 1 representing the best and rank n representing the worst. 2. Each treatment has a sum of 1.0 for all its possible rank probabilities. 3. The darkness of each bar represents the effectiveness of the corresponding treatment, with darker shades signifying better effectiveness. ACU = acupuncture therapy, ASD = routine arthroscopic subacromial decompression, COR = corticosteroid injection, DF = diacutaneous fibrolysis therapy, EXE = routine exercise treatment, HYA = hyaluronate injection, KT = kinesio taping therapy, LLLT = low-level laser therapy, MAN = manual therapy, MWD = microwave diathermy therapy, NON = no treatment/placebo, NSAID = nonsteroidal anti-inflammatory drug injection, onlyBUR = arthroscopic bursectomy without acromioplasty, OSD = open subacromial decompression, PEMF = pulsed electromagnetic field therapy, PLG = platelet-leukocyte gel injection, rESWT = radial extracorporeal shockwave therapy, RF = radiofrequency therapy, SE = specific exercise therapy, US = ultrasound therapy.

Rank probability indicated the possibility of each treatment being the best, the second best, and so forth down to the worst treatment. Figure 5 shows the probability of each rank, whereby each treatment had a sum of 1.0 for all of its possible rank probabilities, and the darkness of the bar represents the effect, with darker colors signifying better results. Figure 6   shows which treatment had the greatest possibility of being the most efficacious treatment based on an analysis of the area under the SUCRA curve, which was drawn according to the cumulative probabilities, with the percentage of the area under each curve shown (larger area signifying a better result).

**FIGURE 6:**
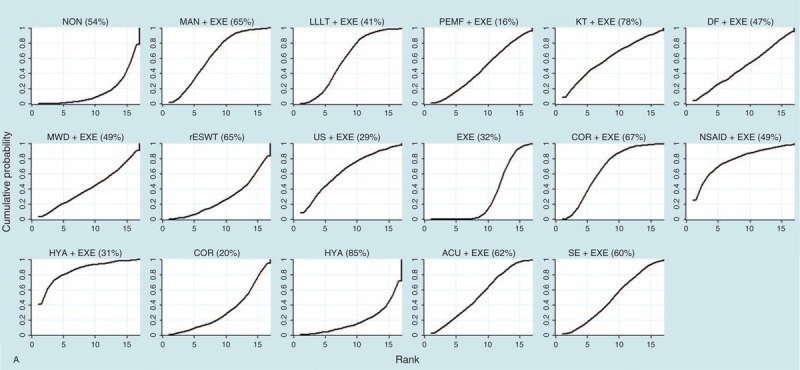
(A) SUCRA for nonoperative treatments (pain score). (B) SUCRA for nonoperative treatments (CMS). (C) SUCRA for operative treatments (pain score). (D) SUCRA for operative treatments (CMS). Note: The area under the curve represents the cumulative rank probability of each treatment, with larger areas signifying higher probabilities. ACU = acupuncture therapy, ASD = routine arthroscopic subacromial decompression, COR = corticosteroid injection, DF = diacutaneous fibrolysis therapy, EXE = routine exercise treatment, HYA = hyaluronate injection, KT = kinesio taping therapy, LLLT = low-level laser therapy, MAN = manual therapy, MWD = microwave diathermy therapy, NON = no treatment/placebo, NSAID = nonsteroidal anti-inflammatory drug injection, onlyBUR = arthroscopic bursectomy without acromioplasty, OSD = open subacromial decompression, PEMF = pulsed electromagnetic field therapy, PLG = platelet-leukocyte gel injection, rESWT = radial extracorporeal shockwave therapy, RF = radiofrequency therapy, SE = specific exercise therapy, US = ultrasound therapy.

**FIGURE 6 (Continued):**
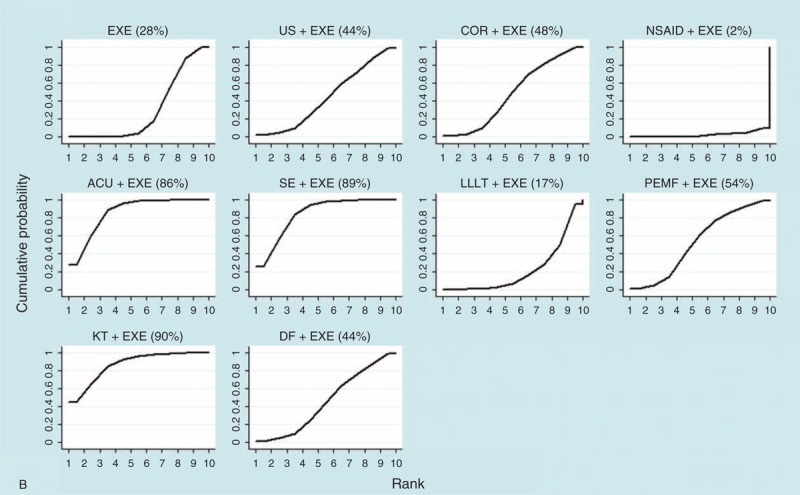
(A) SUCRA for nonoperative treatments (pain score). (B) SUCRA for nonoperative treatments (CMS). (C) SUCRA for operative treatments (pain score). (D) SUCRA for operative treatments (CMS). Note: The area under the curve represents the cumulative rank probability of each treatment, with larger areas signifying higher probabilities. ACU = acupuncture therapy, ASD = routine arthroscopic subacromial decompression, COR = corticosteroid injection, DF = diacutaneous fibrolysis therapy, EXE = routine exercise treatment, HYA = hyaluronate injection, KT = kinesio taping therapy, LLLT = low-level laser therapy, MAN = manual therapy, MWD = microwave diathermy therapy, NON = no treatment/placebo, NSAID = nonsteroidal anti-inflammatory drug injection, onlyBUR = arthroscopic bursectomy without acromioplasty, OSD = open subacromial decompression, PEMF = pulsed electromagnetic field therapy, PLG = platelet-leukocyte gel injection, rESWT = radial extracorporeal shockwave therapy, RF = radiofrequency therapy, SE = specific exercise therapy, US = ultrasound therapy.

**FIGURE 6 (Continued):**
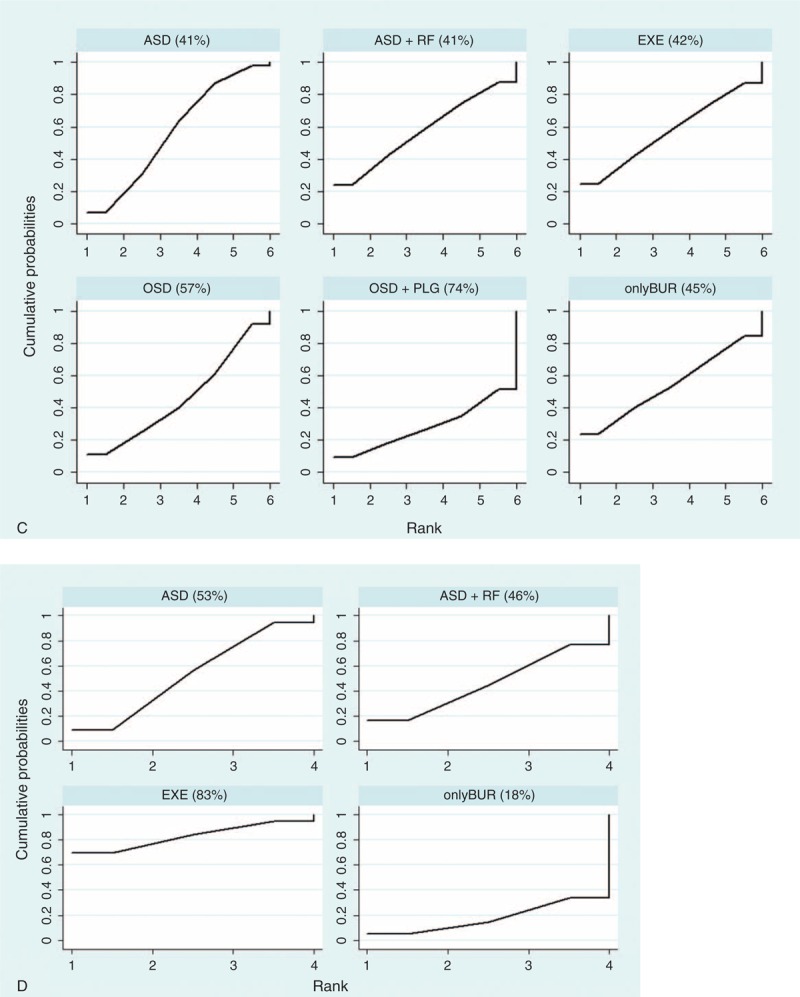
(A) SUCRA for nonoperative treatments (pain score). (B) SUCRA for nonoperative treatments (CMS). (C) SUCRA for operative treatments (pain score). (D) SUCRA for operative treatments (CMS). Note: The area under the curve represents the cumulative rank probability of each treatment, with larger areas signifying higher probabilities. ACU = acupuncture therapy, ASD = routine arthroscopic subacromial decompression, COR = corticosteroid injection, DF = diacutaneous fibrolysis therapy, EXE = routine exercise treatment, HYA = hyaluronate injection, KT = kinesio taping therapy, LLLT = low-level laser therapy, MAN = manual therapy, MWD = microwave diathermy therapy, NON = no treatment/placebo, NSAID = nonsteroidal anti-inflammatory drug injection, onlyBUR = arthroscopic bursectomy without acromioplasty, OSD = open subacromial decompression, PEMF = pulsed electromagnetic field therapy, PLG = platelet-leukocyte gel injection, rESWT = radial extracorporeal shockwave therapy, RF = radiofrequency therapy, SE = specific exercise therapy, US = ultrasound therapy.

For nonoperative treatments, when the outcome was measured by the pain score, HYA+EXE and NSAID+EXE showed better treatment effects than the other treatments, whereas HYA and NON exhibited the worst effects. However, with respect to CMS, a contradictory result was found regarding NSAID+EXE, namely, it demonstrated a worse effect than any other treatment. In this analysis, KT+EXE, SE+EXE, and ACU+EXE were found to be the preferred treatments.

Regarding operative treatments, with respect to the pain score and the CMS, ASD together with treatments derived from it, such as ASD+RF and onlyBUR, showed better effects than OSD and OSD+PLG. Additionally, onlyBUR appeared inferior to ASD and ASD+RF.

### Inconsistency Analyses

In network 1, 1 quadrilateral loop (EXE vs ACU+EXE vs COR vs NON) and 2 triangle loops (EXE vs LLLT+EXE vs US+EXE and HYA vs COR vs NON) were found, but the latter triangle loop (HYA vs COR vs NON) was disregarded because it was described by only one 3-arm trial and no inconsistency was detected. In network 2, 1 triangle loop (EXE vs LLLT+EXE vs US+EXE) was found. There was no loop in networks 3 and 4. In these 3 loops, the Z values were 1.60, 0.01, and 0.01, with corresponding *P* values of 0.11, 0.99, and 0.99, respectively. The *P* values were all >0.05, which demonstrated that no inconsistency was detected in these loops.

### Sensitivity Analyses and Meta-Regression

After the low-quality study^5^ was excluded, the rank probabilities were calculated again. With respect to the pain score, the order of MWD+EXE and PEMF+EXE, which ranked 11 and 12, respectively, was reversed, although the differences between them were very small in both conditions. The ranks of the other treatments remained the same. With regard to CMS, the order of treatment efficacy remained unchanged after the exclusion.

Due to the small number of studies in networks 3 and 4, the meta-regression was only performed for networks 1 and 2. In the meta-regression, no significant difference in the DIC was observed (109.8 and 111.3 for the pain score; 143.6 and 142.1 for CMS), and the 95% CIs of the regression coefficients were −0.04 to 0 for the pain score and −0.08 to 0.32 for CMS, indicating that the covariate (the sample size of the study) was not associated with the treatment effect.

### Summary of Results

The results of pair-wise meta-analysis and network meta-analysis were in good accordance with each other. In terms of nonoperative treatments, exercise-based therapies demonstrated better treatment effects. Regarding operative treatments, the arthroscopic technique tended toward better efficacy than the open surgical technique. These results were supported by inconsistency test, sensitivity test, and meta-regression.

## DISCUSSION

### Advantages and Strengths

This is the first network meta-analysis to include all the available treatment strategies for SIS. It was based on a Bayesian framework and summarized a series of treatment options for SIS from related RCTs, and it was conducted to simultaneously compare various treatment options that have never been directly compared previously. This method overcomes the significant shortcoming of conventional meta-analyses, which cannot compare each treatment versus all other treatment options. The outcome is robust because the prospective design of all the included studies may minimize the selection bias and recall bias. Furthermore, all the included studies were RCTs, which provide the most ideal type of evidence for inclusion in meta-analyses. The sensitivity analysis demonstrated no significant change in the rank probability, with meta-regression also showing no positive findings and the inconsistency analysis showing that all the *P* values were >0.05. Therefore, the outcome of this meta-analysis appears convincing.

### Results for Nonoperative Treatment Options

With respect to the pain score, the results provide support for the effectiveness of exercise therapy. Additionally, treatment options composed of exercise plus other therapies all exhibited a trend toward better effects than exercise alone. These therapies included some common modalities, such as specific exercises, kinesio taping, low-level laser therapy, radial extracorporeal shockwave therapy, and manual therapy, as well as some therapies that are less frequently used, such as acupuncture, diacutaneous fibrolysis, pulsed electromagnetic field therapy, and microwave diathermy ultrasound therapy. However, for localized drug injection therapy, the results appeared to change according to whether exercise therapy was involved; specifically, localized drug injections that were combined with exercise showed better treatment effects than any other treatment options, whereas the worst effects were obtained when they were used alone. Notably, however, no significant difference was found in this set of results due to a wide CI. Only a trend toward better or worse outcomes could be observed.

With respect to the CMS, fewer treatment options were compared due to the limitations of the published data from the included RCTs. However, similar outcomes to the pain score outcomes were observed. Treatment options that were composed of exercise plus other therapies usually yielded better effects than exercise alone. Regarding kinesio taping, specific exercise, and acupuncture therapies, the combined treatment option superiority was supported by significant differences. Additionally, regarding pulsed electromagnetic field, diacutaneous fibrolysis, and ultrasound therapies, only a trend toward a benefit could be obtained. Low-level laser therapy demonstrated a different result from those of other physiotherapies by showing a relatively worse effect than exercise therapy when it was combined with exercise, although this difference had a 95% CI that covered the null value. For treatment options that combined localized injection of NSAIDs and exercise therapy, the CMS results were quite different from the pain score results; specifically, these treatment options exhibited significant inferiority compared with exercise therapy alone. This difference may have been due to the pharmacological properties of NSAIDs. As commonly used analgesic medication, NSAIDs may effectively relieve the sense of pain. However, the CMS evaluation system contains some items besides the pain score. Thus, different outcomes were obtained.

Recently, other studies that were focused on nonoperative treatment options have also been published. Some studies reproduced the effectiveness of exercise,^60^ whereas others found that several treatments may provide additional benefits to an exercise-based regimen,^53^ such as localized injection of corticosteroids^54,61^ and manual therapy.^58^ Some authors demonstrated that no significant difference could be found between specific exercises and extracorporeal shock-wave therapy,^55^ whereas high-level laser therapy demonstrated a better effect than ultrasound therapy.^51^ Some authors reported that acupuncture^56^ and motor control training of the scapula^49^ were more efficacious than ultrasound therapy when applied in addition to exercises.

Some reviews concluded that exercise therapy was effective^64–67^ and that kinesio taping therapy had a small beneficial effect,^68^ whereas no evidence supported the beneficial effects of ultrasound, low-level laser, and electromagnetic field therapies.^69^ In another review,^70^ the authors concluded that ultrasound and extracorporeal shock-wave therapy did not provide additional effectiveness and that exercise resulted in a better effect when combined with manual therapy. Moreover, most of these findings are supported by our study.

### Results of Operative Treatment Options

Compared with nonoperative treatments, fewer options are available for operative treatments. The most commonly used methods were ASD and OSD, which represent arthroscopic and open techniques, respectively. Additionally, certain modified methods were derived from these modalities, such as ASD combined with radiofrequency, ASD without acromioplasty, and localized injection of platelet-leukocyte gel combined with OSD.

In this meta-analysis, no significant difference in the treatment effect was detected with respect to either the pain score or the CMS. However, the arthroscopic technique tended toward better efficacy than the open surgical technique. Furthermore, acromioplasty may play an important role in the arthroscopic technique to some extent because bursectomy without acromioplasty does not appear to be as good as standard ASD and ASD combined with radiofrequency. Another notable finding was that exercise therapy also demonstrated an excellent effect in this subgroup.

Some reviews and other studies regarding these techniques have been published in the past few years. In some published RCTs, the authors reported that the use of radiofrequency^59^ and laser^57^ therapy provided no additional benefit to ASD. In 1 study, the authors emphasized that OSD was equivalent to ASD at the 1-year follow-up.^33^ In other studies, the authors concluded that ASD had a better short-term effect because patients could spend less time in the hospital and could return to their activities of daily living and work more quickly^62^; however, after 1 year, the OSD group tended to catch up, although ASD still showed significant benefits in some respects.^37^ Some authors have concluded that the difference between ASD and supervised exercise is not clinically important^63^ and that supervised exercise should be the basis of treatment for SIS.^52^ A systematic review, which focused on the comparison between standard ASD and bursectomy only, concluded that there was no significant difference between them.^71^ Another review concluded that ASD and OSD had equivalent ultimate clinical outcomes.^72^ According to another systematic review, there was no evidence that a certain surgical treatment option was better than another or better than nonoperative treatment option.^73^ Moreover, these findings are also supported by our study.

## LIMITATIONS

Certain limitations existed in this meta-analysis. First, most included RCTs had brief follow-up periods, most of which lasted <1 year, and further studies with longer follow-up periods may be required to support our conclusions. Second, although no significant inconsistency was found by the Z test, we recognize that the number of loops was only 3 (2 in network 1 and 1 in network 2). This limitation may be resolved if additional head-to-head trials are included in future studies. Third, most comparisons were performed based on only 1 RCT, so the potential for bias should not be neglected. This problem could be solved by replicating the RCTs in the future. Fourth, the insufficient blinding of most studies may have caused potential bias in the assessment of treatment effects.

## CONCLUSIONS

Exercise and other exercise-based therapies are the most important treatment options for SIS patients. For those patients who seek nonoperative treatment option at an early stage of SIS, exercise combined with other therapies should be recommended. Among these therapies, kinesio taping, specific exercises, and acupuncture therapy should be considered as the first-line choices, whereas pulsed electromagnetic field therapy, localized corticosteroid injection, diacutaneous fibrolysis, and ultrasound therapy may be considered as the second-line treatment choices; however, low-level laser therapy and the localized injection of NSAIDs are not recommended. For patients with chronic SIS, operative treatment options may be considered. In this case, standard arthroscopic subacromial decompression surgery is a relatively superior option to open subacromial decompression and arthroscopic bursectomy. Notably, however, the decision for operative treatment should be made cautiously because similar outcomes may also be achieved by the implementation of exercise therapy.
