# Treatments for Shoulder Impingement Syndrome: A PRISMA Systematic Review and Network Meta-Analysis: Erratum

**DOI:** 10.1097/01.md.0000484495.36196.d5

**Published:** 2016-06-10

In the article “Treatments for Shoulder Impingement Syndrome: A PRISMA Systematic Review and Network Meta-Analysis”,^1^ which appeared in Volume 94, Issue 10 of *Medicine*, Figure 6A and 6C contained errors. The article has since been corrected online.
